# Effects of Enzyme Replacement Therapy Started Late in a Murine Model of Mucopolysaccharidosis Type I

**DOI:** 10.1371/journal.pone.0117271

**Published:** 2015-02-03

**Authors:** Gabriela Pasqualim, Guilherme Baldo, Talita Giacomet de Carvalho, Angela Maria Vicente Tavares, Roberto Giugliani, Ursula Matte

**Affiliations:** 1 Post-Graduation Program in Genetics and Molecular Biology (PPGBM), Universidade Federal do Rio Grande do Sul, Porto Alegre, Brazil; 2 Gene Therapy Center, Experimental Research Center, Hospital de Clínicas de Porto Alegre, Porto Alegre, Brazil; 3 Department of Physiology, Instituto de Ciências Básicas da Saúde, Universidade Federal do Rio Grande do Sul, Porto Alegre, Brazil; 4 Health Science Department, Uniritter—Laureate International Universities, Porto Alegre, Brazil; 5 Medical Genetics Service, Hospital de Clínicas de Porto Alegre, Porto Alegre, Brazil; 6 INAGEMP, Porto Alegre, Porto Alegre, Brazil; 7 Department of Genetics, Instituto de Biociências, Universidade Federal do Rio Grande do Sul, Porto Alegre, Brazil

## Abstract

Mucopolysaccharidosis type I (MPS I) is a progressive disorder caused by deficiency of α-L-iduronidase (IDUA), which leads to storage of heparan and dermatan sulphate. It is suggested that early enzyme replacement therapy (ERT) leads to better outcomes, although many patients are diagnosed late and don’t receive immediate treatment. This study aims to evaluate the effects of late onset ERT in a MPS I murine model. MPS I mice received treatment from 6 to 8 months of age (ERT 6–8mo) with 1.2mg laronidase/kg every 2 weeks and were compared to 8 months-old wild-type (Normal) and untreated animals (MPS I). ERT was effective in reducing urinary and visceral GAG to normal levels. Heart GAG levels and left ventricular (LV) shortening fraction were normalized but cardiac function was not completely improved. While no significant improvements were found on aortic wall width, treatment was able to significantly reduce heart valves thickening. High variability was found in behavior tests, with treated animals presenting intermediate results between normal and affected mice, without correlation with cerebral cortex GAG levels. Cathepsin D activity in cerebral cortex also did not correlate with behavior heterogeneity. All treated animals developed anti-laronidase antibodies but no correlation was found with any parameters analyzed. However, intermediary results from locomotion parameters analyzed are in accordance with intermediary levels of heart function, cathepsin D, activated glia and reduction of TNF-α expression in the cerebral cortex. In conclusion, even if started late, ERT can have beneficial effects on many aspects of the disease and should be considered whenever possible.

## Introduction

Mucopolysaccharidosis type I (MPS I) is a rare autosomal recessive disorder caused by deficiency of lysosomal hydrolase alpha-L-iduronidase (IDUA, EC 3.2.1.76), involved in the degradation of glycosaminoglycans (GAG) heparan sulphate (HS) and dermatan sulphate (DS). Its deficiency leads to progressive accumulation of undegraded or partially degraded substrate within lysosomes, with subsequent multiorgan dysfunction and damage [1]. There is a considerable clinical variability in the age of onset and rate of disease progression [2]. However, three classical phenotypes are usually considered: severe Hurler (OMIM #67014), intermediary Hurler Scheie (OMIM #607015) and the attenuated Scheie syndrome (OMIM # 67016) [3].

The Hurler form corresponds to 50–80% of known cases [2]. It shares many systemic manifestations that are found in the attenuated forms, such as growth retardation hepatosplenomegaly, joint stiffness, heart disease and respiratory insufficiency. However, it is rapidly progressive, presents progressive neurodegeneration and death usually occurs during the first decade of life [1,4,5].

Two treatment options are available for MPS I: hematopoietic stem cell transplantation (HSCT) and enzyme replacement therapy (ERT). HSCT is the treatment of choice for Hurler patients and when performed before cognitive impairment begins, it can significantly preserve intellectual development [2,6,7]. ERT with laronidase (Aldurazyme, Genzyme Corporation) has been approved for human use since 2003/2005 (USA and Europe/Brazil). It is indicated for treatment of the non-neurological symptoms of MPS I [2,6,8–10]. The most constant results from laronidase clinical trials are decrease of urinary GAG excretion, and reduction of hepatosplenomegaly and apnea/hypopnea episodes, regardless of the patient’s clinical form. Moreover, improvements in 6-Minute Walk Test [4,11], increase in height and weight growth rate [12], increase in shoulder and/or elbow range of flexion [4,12], and stabilization or improvement in forced vital capacity [4,11] were also observed.

There is a consensus in the literature that, being a progressive disorder, early treatment leads to a better outcome of MPS I patients, preventing or minimizing irreversible damage [2,4,6,9,13–16]. However, worldwide many patients are diagnosed later in life and do not receive immediate treatment. Therefore, the aim of this study was to evaluate the effects of ERT when started later in life on the reversibility of established disease manifestations in a murine model of MPS I.

## Material and Methods

### Experimental Groups

*Idua* ^-/-^ mice on a C57BL/6 background [17]; kindly donated by Dr Elizabeth Neufeld (UCLA, USA), and their normal (*Idua* ^+/+^ or *Idua* ^+/-^) littermates were used. Animals were maintained at 20°C with food and water *ad libitum*. MPS I (*Idua* ^-/-^) and normal mice were genotyped by PCR as previously described [18].

ERT was introduced at 6 months of age (adult mice). MPS I mice started receiving 1.2mg/kg of laronidase (Aldurazyme, Genzyme) intravenously every two weeks until 8 months of age (ERT 6–8 mo, n = 10). Wild-type and untreated 8 month-old mice were used as control groups (Normal, n = 8 and MPS I, n = 11). Animals from both genders were used in all groups. Five males and 5 females were used in the treated group. In the control groups, approximately the same numbers of animals from both sexes were used. Throughout our study, gender comparisons were performed in each analysis to ensure that threre were no significant differences between sexes.

The enzyme dose and regimen was the same used in a previous study from our group [19]. All procedures performed in this study are summarized in S1 Fig.

### Tissue collection and histological analysis

At 8.5 months old (two weeks after last enzyme infusion in ERT group) mice were anesthetized with isoflurane, serum was collected by retro-orbital puncture and mice were euthanized by cervical dislocation. Liver, kidneys, lungs, heart, aorta, and the brain (cerebellum and cortex) were isolated and divided in two parts. One was frozen in a-80°C freezer for biochemical analysis and the other portion was fixed in buffered formalin.

Paraffin processing was performed according to routine techniques. Thin cross sections were submitted to routine histological processing, stained with Hematoxylin-Eosin and Alcian Blue and analyzed. Heart valves from the left ventricle were obtained by sectioning the basal part of the heart, and valve thickness was measured for at least 3 different fields in at least 4 points each using the QCapture software (Q Imaging, British Columbia, Canada), from which average was calculated and used as a measure of valve thickness. Immunohistochemistry for glial fibrillary acidic protein (GFAP) was performed in cerebral cortex using a specific antibody (Dako, Denmark) according to manufacturer’s instructions. Slides were analyzed counting positive cells in 5 fields (400X magnification).

### GAG measurement

Frozen tissues were homogenized in 0.5 mL phosphate buffer 50 mM pH 6.5 with 0.24 g/L L-cysteine and 0.4% EDTA 0.5 M, incubated overnight at 60°C, mixed with 150 μL chloroform and centrifuged for 15 minutes at 10000G at 4°C. GAGs were measured using the dimethyl blue (DMB) technique, in which 25 μL of supernatant is mixed with freshly prepared DMB solution (DMB 0.3 mol/L with 2 mol/L Tris) and absorbance was read at 530 nm. For cerebellum and cerebral cortex, 125 μL were used. Results were normalized by protein quantity and expressed as percentage of normal mice.

Urine samples were centrifuged and 25 μL were used for measuring GAG levels. Results were normalized with creatinine, which was measured using the Picric acid method [20].

### Echocardiographic analysis

Eight-month old mice were anesthetized with isoflurane and placed in left lateral decubitus position (45° angle) to obtain cardiac images. An EnVisor HD System, Philips Medical (Andover, MA, USA), with a 12–4 MHz transducer was used, at 2 cm depth with fundamental and harmonic imaging. Images were captured by a trained operator with experience in small animal echocardiography. All measurements were performed as previously described [20]. Briefly, equations used were: Left Ventricular Ejection Fraction (LVEF) = (end diastolic volume− end-systolic volume/ end-diastolic volume) × 100. LV fraction shortening (LVFS) values: LVFS = (diastolic diameter − systolic diameter) / diastolic diameter × 100. Fractional area change (FAC) = diastolic area—systolic area / diastolic area. Measures of the ejection and acceleration times in the pulmonary valve were obtained using Doppler echocardiography, and their ratio (AT/ET) was used as an index of pulmonary vascular resistance [21].

### Open Field test

Locomotor and exploratory activities were assessed by open field test in an Activity Monitor IR equipment (Insight, São Paulo, Brazil). It consists of a closed box with area of 50 x 50 cm and clear walls. The floor is divided in 64 squares and it has 16 electronic sensors in each side. Mice were placed in one of the corners of the open field and crossings, rearings, distance moved and velocity were analyzed for 5 minutes. All tests were performed by the same person at the same period of the day in the same room, which was free of noise or other interferences.

### Cathepsin D activity

Cerebral cortex was homogenized in 100 mM sodium acetate buffer pH 5.5 containing 2.5 mM ethylenediaminetetraacetic acid, 0.1% Triton X-100, and 2.5 mM dithiothreitol. The cathepsin D (CtsD) assay was performed as described by Baldo et al. [22] at pH 4 with 5 μM of the substrate 7-methoxycoumarin-4-acetyl (Mca)-Gly-Lys-Pro-Ile-Leu-Phe-Phe-Arg-Leu-Lys-2,4 nitrophenyl (Dnp)-D-Arg-NH2, which can also be cleaved by CtsE. The fluorogenic peptide Mca-Pro-Leu-OH (Enzo Life Sciences, USA) was used as the standard.

### Antibody formation

Serum IgG anti-laronidase antibodies were measured at time of euthanasia as previously described [20]. Briefly, 96-well ELISA plates were coated with 4 μg/mL of laronidase (Aldurazyme, Genzyme Corporation) in acid PBS overnight at 4°C. Then, it was blocked with acid PBS 3% BSA and diluted serum was added (1:50 for normal and MPS I controls and 1:250 for treated mice) in duplicates and incubated for 2h. The secondary antibody conjugated to peroxidase (Goat anti-mouse IgG, Sigma, USA) was diluted 1:1000, incubated for 3h and revealed with TMB for 3 minutes. The reaction was stopped with sulfuric acid 2 N and the absorbance was read at 450 nm.

### qRT-PCR

RNA was extracted from frozen cerebral cortex with Trizol Reagent (Invitrogen, USA) according to the manufacturer's instructions. Reverse transcription (RT) was performed with High Capacity cDNA Reverse Transcription Kit followed by 20 μl qRT-PCR reactions with SYBR GreenER qPCR SuperMix Universal (Life Technologies, USA). mRNA expression levels of Macrophage inflammatory protein-1α (MIP1-α or CCL3) and Tumor necrosis factor (TNF-α) were normalized by expression of Glyceraldehyde 3-phosphate dehydrogenase (GAPDH). Reactions were performed in triplicates in standard conditions with annealing temperature of 60°C for all genes in a Mx3000P qPCR System (Stratagene,USA). Primer sequences used were: GAPDH, 5’CCCATCACCATCTTCCAGG3’ and 5’CATATTTGGCAGCTTTCTCC3; MIP1-α, 5’CTGCAACAAAGTCTTCTCA3’ and 5’GACTTCAGTTTCAGGTCAGT3’ and TNF-α, 5’CATCTTCTCAAAATTCGAGTGACAA3’ and 5’TGGGAGTAGACAAGGTACAACCC3’.

### Ethics Statement and Statistics

This study was approved by the authors' institutional ethics committee on animal experimentation (Comissão de Ética no Uso de Animais do Hospital de Clínicas de Porto Alegre) and all experiments with animals were monitored by a veterinary.

IBM SPSS Statistics version 20 was used for statistical analysis. Possible gender effects were analyzed in all tests, and unless otherwise stated, no significant differences were found between males and females. Results were compared using ANOVA and Tukey or Kruskall-Wallis and Mann-Whitney, as indicated. P values lower than 0.05 were considered statistically significant. GraphPad Prism 5 software was used to graphic design.

## Results

### GAG levels

**Fig 1:**
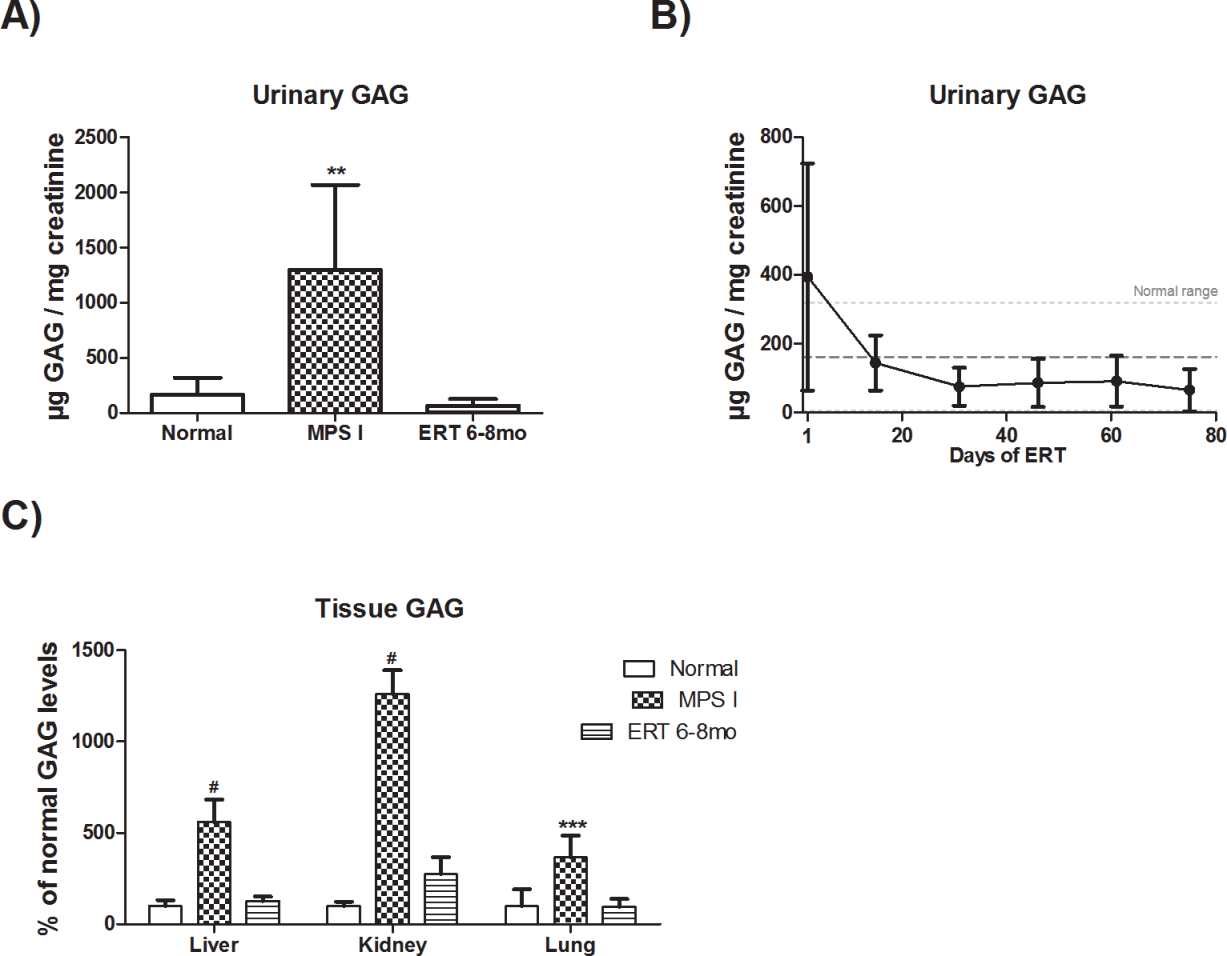
Urinary and tissue GAG levels. A) MPS I mice treated from 6 to 8 months with 1.2mg laronidase/kg every two weeks (n = 10). Urine was collected 1 day post injections. **P<0.01, ANOVA and Tukey. B) Comparison between urinary GAG from 8-month old wild-type (normal, n = 4), MPS I (n = 3) and treated MPS I mice (ERT 6–8mo, n = 10), 1 day after last injection. Treated mice achieve normal GAG level (dotted lines) as soon as the second injection. C) Tissue GAG from 8-month old normal (n = 4/5), MPS I (n = 5–6) and ERT 6–8mo (n = 10) mice collected two weeks after last injection. #P<0.05, Kruskal-Wallis and Mann-Whitney *post hoc*. ***P<0.001 ANOVA and Tukey *post hoc*.

Urinary GAG showed a marked and rapid reduction, with treated MPS I mice reaching near normal levels after the second laronidase injection (Fig. 1A). At the end of treatment, 6–8 month old ERT-treated mice showed levels significantly lower than untreated 8 month MPS I and equal to that of normal mice (Fig. 1B, P<0.01, ANOVA and Tukey).

Eight month-old MPS I mice presented liver, kidney and lung GAG levels approximately 5, 12 and 4 times higher than normal mice, respectively (Fig. 1C). ERT started at 6 months significantly reduced storage in these organs, reducing it to normal range (P<0.05 for liver and kidney; P<0.001 for lungs).

### Heart function

**Fig 2:**
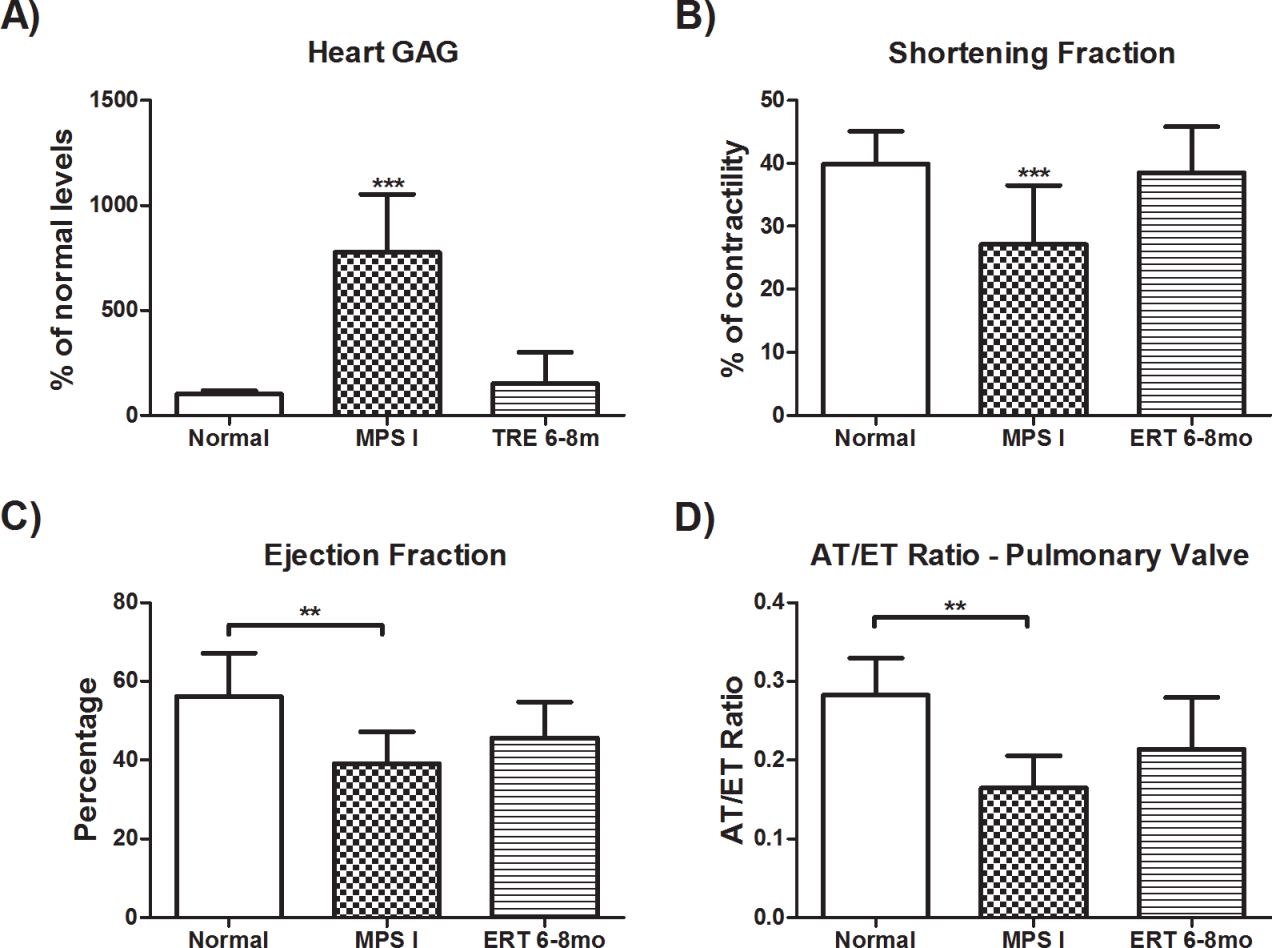
Heart function. A) GAG storage in hearts from 8-month old normal (n = 3), MPS I (n = 5) and MPS I mice treated with ERT from 6 to 8 months (ERT 6–8mo, n = 10), 2 weeks after last injection. B-D: Echocardiography results from 8-month old normal (n = 6/8), MPS I (n = 6/10) and MPS I mice treated with ERT from 6 to 8 months (ERT 6–8mo, n = 10), 1 day after last injection. B) Ejection fraction, C) Shortening fraction D) Ratio between ejection and acceleration times measured at the pulmonary valve. ***P≤0.001, **P≤0.006 ANOVA and Tukey *post hoc*.

GAG storage in the hearts of untreated mice was nearly 8 times higher than normal range. ERT started late was able to revert this situation (P<0.001) (Fig. 2A), which could lead to a better heart function in treated mice. Echocardiography analysis revealed a significant improvement of LV shortening fraction in the ERT 6–8mo group, when compared to untreated mice (P≤0.001, Fig. 2B). Regarding LV Ejection Fraction and the Acceleration and Ejection Ratio at the pulmonary valve no significant difference was found between treated and untreated animals although a trend towards improvement was seen (Fig. 2C and 2D).

### Heart Valves and Aorta

**Fig 3:**
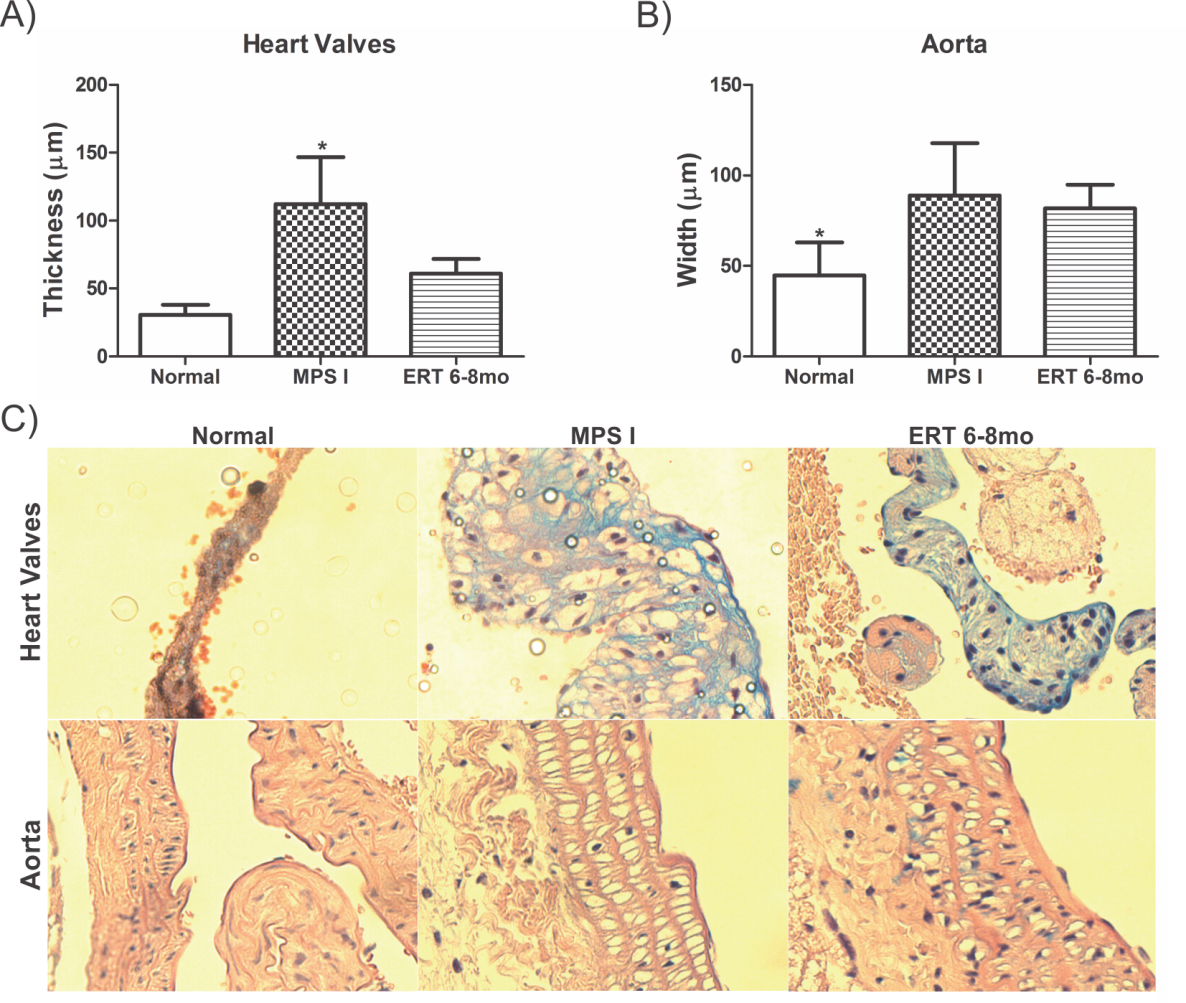
Heart valves and Aorta. A) Heart valve thickness and B) Aortic wall width in 8-month old normal (n = 3/4), MPS I (n = 3/5) and 6 to 8 months laronidase treated MPS I (ERT 6–8mo, n = 5/10), 2 weeks after last injection. *P≤0.03, ANOVA and Tukey *post hoc*. C) Heart valves and aortas stained with H-E and Alcian Blue. The presence of large vacuoles and intense alcian blue staining indicating GAG storage are evident in thick MPS I mice valves but are significantly reduced in treated animals. A large amount of vacuoles are also seen in the dilated MPS I and ERT 6–8mo aortas. Original magnification, ×400.

Laronidase treatment resulted in significant reduction in heart valves thickness when compared to untreated mice: 112.01±34.3 μm in MPS I versus 61.1±10.8 μm in treated mice (mean ± SD, P≤0.03) (Fig. 3A). As also shown in histological sections (Fig. 3C), the presence of large vacuoles and intense alcian blue staining indicating GAG storage were evident in thick MPS I mice valves slides and significantly reduced in treated animals, although some vacuoles could still be observed.

Aortic walls from MPS I mice presented twice the width of normal mice (Fig. 3B). ERT started at 6 months was not able to normalize this aspect of the disease. Normal mice aorta width was significantly lower than both treated and untreated MPS I animals. Fig. 3C, bottom row, shows the large amount of vacuoles that were also seen in the dilated aortas from MPS I and ERT 6–8mo mice.

### Open Field test

**Fig 4:**
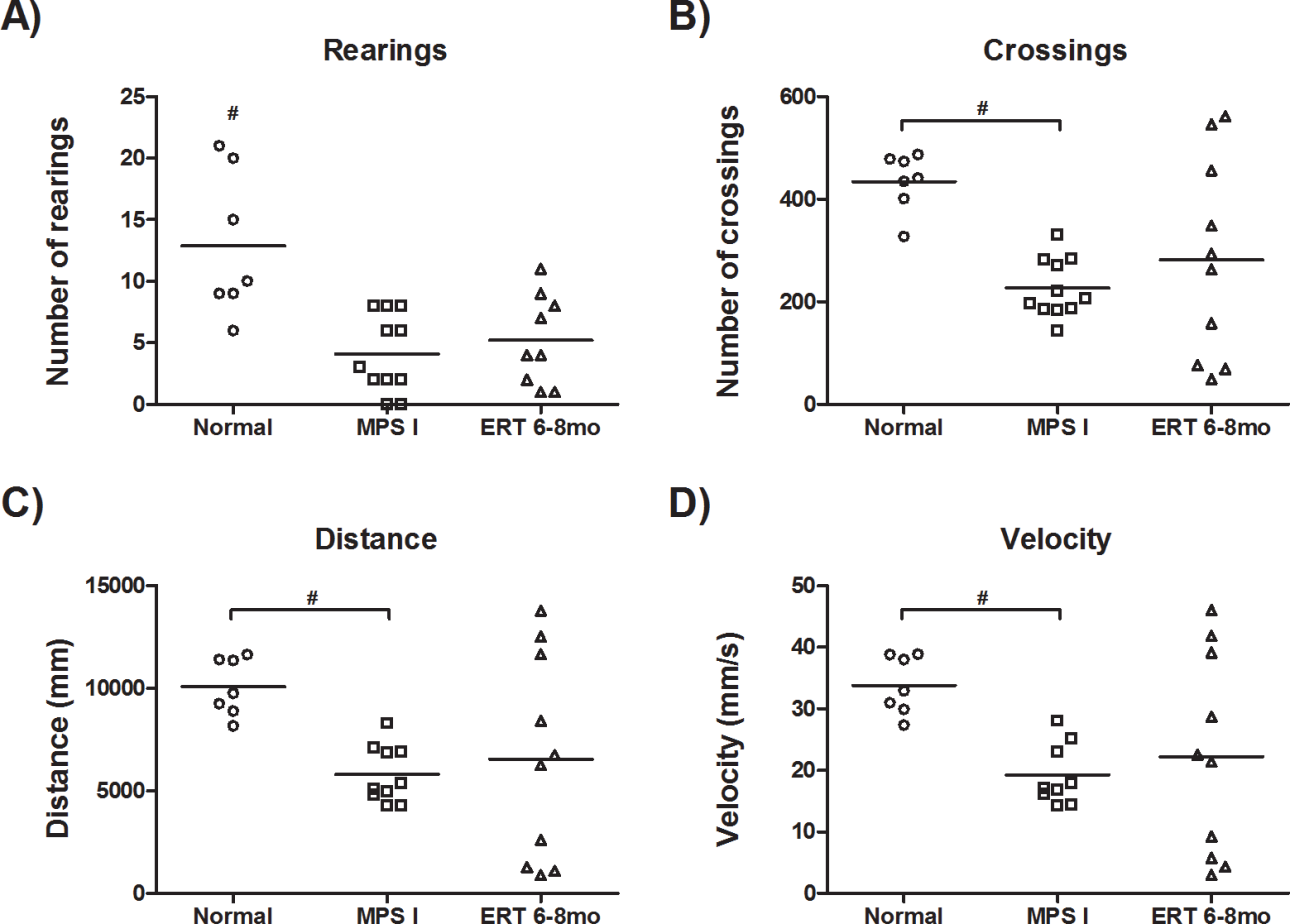
Open field test. Results from 8-month old normal (n = 7), MPS I (n = 11) and 6 to 8 months laronidase treated MPS I (ERT 6–8mo, n = 10), 2 weeks after last injection. #P≤0.04, Kruskal-Wallis and Mann-Whitney *post hoc*.

Exploratory behavior (measured as the number of rearings, Fig. 4A) was significantly lower in both treated and untreated mice when compared to normal animals (P≤0.04). The ERT 6–8mo group presented intermediate values without statistical significance between normal and MPS I mice in all other characteristics analyzed: number of crossings, velocity and distance walked (Fig. 4B-D). Interestingly, this test showed the highest variability among treated animals compared to all other measurements performed in this study.

### Cerebral GAG levels and Cathepsin D activity

**Fig 5:**
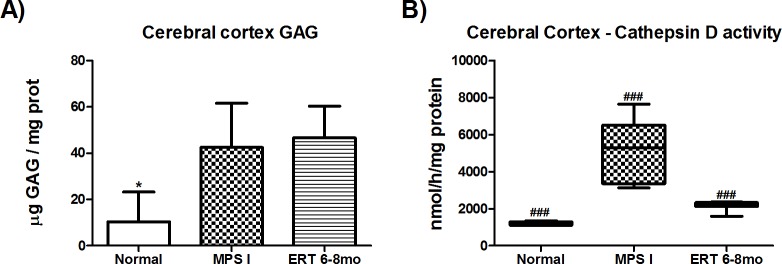
Brain disease markers. A) Cerebral cortex GAG levels and B) Cathepsin D activity in 8-month old normal (n = 5), MPS I (n = 5) and 6 to 8 months laronidase treated MPS I (ERT 6–8mo, n = 10), 2 weeks after last injection. *P = 0.02, ANOVA and Tukey. ###P<0.001, Kruskal-Wallis and Mann-Whitney *post hoc*.

In order to explain the large variation found in the ERT 6–8mo group behavior, we measured GAG levels in the cerebral cortex from all groups. ERT treatment had only minimal effect on cerebral cortex GAG storage. A near 5-fold increase in GAG storage was found in both MPS I and ERT 6–8mo mice cerebral cortex when compared to normal levels (P≤0.02, Fig. 5A).

Cerebral GAG storage was unsuitable to elucidated behavioral heterogeneity. Therefore, cathepsin D activity was measured, as it has been shown as a surrogate marker of enzyme deficiency [22–24]. MPS I animals presented cathepsin activity 4 times greater than normal mice (P<0.001, Fig. 5B). ERT 6–8mo mice had a significant reduction to 1.7-fold normal values (P<0.001, Fig. 5B).

### Neuroinflammation

**Fig 6:**
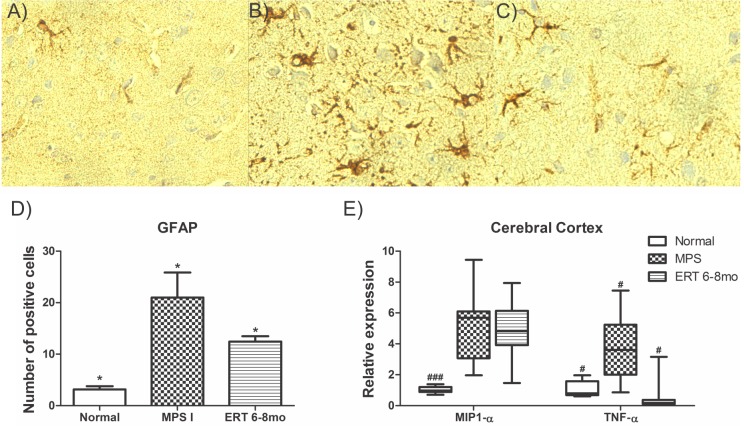
Neuroinflammatory markers. A-C) Representative sections from 8-month normal (A, n = 5), MPS I (B, n = 5) and 6 to 8 months MPS I laronidase treated (C, n = 10) mouse cerebral cortex stained for glial fibrilary acidic protein (GFAP). D) Number of positive cells counted in 5 fields (400× magnification) in mouse cortex at 8 months. *P ≤0.02. ANOVA and Tukey. E) MIP1-α and TNF-α mRNA expression on cerebral cortex normalized by GAPDH expression. ###P≤0.001, #P ≤0.02 Kruskal-Wallis and Mann-Whitney *post hoc*.

Glia activation was analyzed by GFAP immunostaining. Untreated MPS I mice had a 7-fold increase in GFAP-positive cells when compared to normal mice (p<0.001, Fig. 6A-D). Treated mice had a decrease to 4 times the normal range, being intermediary, yet significantly different, from both normal and MPS controls (P≤0.02 Fig. 6A-D). In addition, mRNA expression of macrophage inflammatory protein (MIP-1a/CCL3) and tumor necrosis factor α (TNFα), two proinflammatory cytokines, were analyzed in cerebral cortex. Laronidase treatment had no effect on cortex MIP1-α mRNA expression when compared to untreated mice (Fig. 6E). However, TNF-α levels were significantly decreased (P≤0.001, Fig. 6E).

### Antibody formation

Animals who received ERT treatment had significantly higher levels of serum IgG Anti-laronidase antibodies than normal and untreated MPS I controls (P<0.001, S2 Fig.). However, no correlations were found between antibody formation and behavior tests, GAG levels, cathepsin D activity or neuroinflammatory markers (data not shown).

## Discussion

MPS I is a progressive multisystemic disease and there is a clear consensus in the literature that for both HSCT and ERT early treatment leads to better outcomes. Nonetheless, data from the MPS I Registry show that Hurler patients from Latin America are diagnosed at a later age than the rest of the world and that there is an average delay of 1.9 to 3.1 years between median age of diagnosis and the median age of first treatment for Hurler patients [25,26]. A study done by Vieira et al. (2008) which collected data solely from Brazilian MPS patients showed a great delay in diagnosis, when compared to other reports from the literature: 30 to 75 months. It is important to point out that due to the nature of the public health system in Brazil, medical appointments, particularly with specialists, can take months to be scheduled. In addition, there is a lack of knowledge of Brazilian health professionals about MPS [27]. Another important issue is the shortage of specialists to meet the demand of the population. Currently, there are 172 registered professionals in the Brazilian Society of Medical Genetics, from which 82 are located in São Paulo and Rio de Janeiro, to attend a population of approximately 191 millions of habitants spread across 8.5 billions of km^2^ (data from Brazilian Institute of Geography and Statistics—IBGE). In addition to delayed diagnosis, the access to the medication is also very problematic. Consequently most patients are already older than indicated to undergoing HSCT and to achieve maximal benefits from ERT. Even so, ERT has become the most used treatment in Brazil and other Latin American countries [28]. Therefore we analyzed effects of ERT in adult MPS mice, when symptoms are well established.

Urinary GAG levels in the ERT 6–8mo group were normalized as soon as the second injection and maintained low levels throughout the treatment. In untreated animals there was an expressive increase in GAG levels when compared to previous reports of 6 months MPS animals [19]. It is important to stress that total urinary GAG may not reflect major clinical signs and symptoms and GAG content in urine is thought to reflect primarily storage in renal tubular epithelial cells [29,30]. Consistently whit this observation, kidney storage was higher than previously reported as well [31]. Moreover, ERT started late was also able to normalize storage in other visceral organs as liver and lungs. Weight evaluation showed no statistically significant improvement after treatment (data not shown), which is in accordance to what has been shown previously by our group in animals treated from 2 to 6 months of age [19].

Cardiac manifestations are common in MPS I patients and worsen with age [2]. Heart GAG level presented an 8-fold increase in MPS mice. It was significantly reduced to normal levels in the ERT 6–8mo group. However, this reduction was not completely translated into a better heart function. The LV shortening fraction was normalized and there was also an improvement in the LV ejection fraction, which indicates an enhancement in myocardial contractility. The AT/ET ratio reflects pulmonary vascular resistance and pulmonary hypertension. It was only partly corrected after treatment, suggesting that this aspect of the disease might be harder to correct and/or needs a longer treatment length. In previous experiments, all three parameters could be normalized by ERT administrated with the same dose and periodicity started either in the neo-natal period or from 2 to 6 months of age [19]. Therefore, our data suggest that this dose regimen is able to prevent but not to completely reverse heart disease in the mouse model of MPS I (S1 Table). A report from Gabrielli et al. (2010) on two siblings with attenuated MPS I held similar results. The younger brother, treated from 5 months had no cardiac disease before or after 5 year of laronidase treatment. The older sister treatment’s began at 5 years of age, when she already presented pulmonary hypertension. Five years later, her heart disease was not improved but stabilized.

The most prominent cardiac manifestation in MPS I is valvular abnormality [9]. Although usually not fatal, it may contribute to clinical disease [32]. Therefore we also evaluated heart valves thickening and aortic wall width. In this study, ERT started late had no significant results on aortic width. On the other hand, heart valves in the ERT 6–8mo were about half the size when compared to untreated animals and had no statistical difference from the normal group. Similar results were reported in the canine model. Animals treated weekly with 1.57mg/kg from birth showed valves similar to normal animals and mice treated with 0.58mg/kg still had some residual storage [33]. When adult animals where treated, only the ones with induced immune-tolerance to IDUA had improvement in the valves [34]. In our data, no correlation between immune status and valve thickening was found (data not shown). In addition, studies in humans showed that ERT has no or only minor effects in aortic width and valve thickness once it is established [4,10,13,35–37]. HSCT also has no effect on cardiac valvular thickening progress in some patients [38].

In the usual dose, the infused enzyme is not expected to cross the blood brain barrier (BBB) at therapeutic levels [8], which is the reason why ERT isn’t indicated for neurological manifestations of Hurler patients. Therefore, data on cognitive analysis from Hurler patients after ERT is very scarce. Wraith et al. (2007) reported that Hurler patients treated from less than 2.5 years of age showed mental development similar to normal children, whereas older patients showed no improvement. Tokic et al. (2007) reported that two patients had no improvement in mental state but positive changes were observed in humor and behavior. Similar results are found with HSCT, as cerebral damage present before transplantation seems to be irreversible [39]. However, evidence from animal studies is challenging this idea, suggesting that at least some enzyme is able to cross the BBB [40,41]. A study with the murine metachromatic leukodystrophy model evaluated ERT effects on presymptomatic, early and progressed stages of the disease [42]. ERT efficacy was shown to be age-related, with improvement in behavioral alterations seen only with early treatment. Previous data from our group suggest that early (starting neonatal or at two months) administration of ERT seems effective in preventing or normalizing brain disease in MPS I mice [19]. The animals treated in the present study were adults with well-established cognitive impairment. Therefore, no positive changes on neurologic and musculoskeletal functions were expected. Still the high heterogeneity found in the open field test was remarkable, with some animals showing signs of improvement. It is strikingly that treated animals clearly can be divided into three groups, one with parameters similar to normal mice, the other intermediate and the last similar to untreated affected animals.

We then investigated if this apparent group of mice with “improved” behavior presented other distinctive characteristics. No reduction was observed in cerebral cortex GAG of treated animals, regardless of open field test results. Cathepsin D, possibly a more accurate biomarker, also did not reflect the observed heterogeneity, since treated animals had intermediary values between normal and untreated mice. Heart function, tissue and urinary GAG levels also showed no relation to open field data (data not shown). Bone and joint improvements could also alter the results for the test. However, a previous report from our group [19] found no improvement in joint disease in 6 months old mice neither after neonatal nor adult ERT (2 to 6 months of age). Therefore, we also do not expect improvements after the late treatment applied in this study.

Neuroinflammation has been reported to play a role in the progression of brain disease in MPS I [17,18,43,44]. Glia activation, which suggest a neuroinflammatory process, can be normalized with ERT when stared at birth or at 2 months of age [19]. Our data shows that ERT even started at a late stage of the disease can reduce this process in cortex since treated animals had significantly less GFAP positive cells than untreated 8 month MPS mice. The expression of cytokines MIP1-α and TNF-α were also studied. MIP1-α is a chemokine released by glia which attract monocytes into the CNS and has elevated levels on the brain of MPS I mice [44]. TNF-α is an proinflammatory cytokine released by the activated glia that propagates inflammation and its expression is also increased in MPS mice brain [45]. The reduction of activated glia was only partially reflected by lower levels of proinflammatory cytokines in cortex. MIP1-α mRNA levels from treated mice presented a reduction from untreated controls, although it was not statistically significant. However, TNF-α expression was significantly reduced on treated animals indicating a partial improvement on neuroinflammation. Nevertheless, these changes were not correlated to improved behavior in that subset of animals.

Finally, antibody formation against the enzyme was also investigated as a possible explanation for the behavior results. Almost all patients on clinical trials produce IgG antibodies against laronidase, particularly in the first weeks of treatment [4,10,11,30,46]. An inverse correlation was found between antibody titer and urinary GAG reduction, however no significant correlation was established with clinical efficacy [4,13,30]. Reports from animal models, on the other hand support the idea that antibodies titer decrease the effectiveness of treatment. A study by Dickson et al. (2008) in the canine model showed that immune-tolerized dogs achieved increased tissue enzyme levels in most non reticular tissues and a greater reduction in tissue GAG levels, lysosomal pathology, and urinary GAG excretion when compared to non-tolerized animals. A previous report by our group showed that mice which developed antibodies performed significantly worse in the open field habituation test [19]. However, data presented in this study is not consistent with this hypothesis. Although all treated animals developed antibodies against laronidase, no significant correlations were found between antibody titer and GAG level, behavior tests or cathepsin D activity (data not shown).

It is important to emphasize that the behavioral results found in this study do not perfectly match the previous ones from our group. Although in both cases animals were placed on the same location and the same parameters were analyzed for the same time, the equipment’s used were different. Baldo et al (2012; 2013) utilized a square arena (52 cm × 52 cm) with the floor divided into 16 squares. All measurements were made manually. Here, all measurements were made automatically by the equipment with an area of 50x50cm, divided in 64 squares and 16 sensors in each side. Therefore, in this study, sensitivity for crossings was much higher and the number of events was greater. On the other hand, sensitivity for rearings is higher when counted manually because mice needed to achieve a certain height to be detected by the sensors. Consequently, data from this parameter was lower. Nevertheless, the significant difference found in locomotor and exploratory activities between normal and MPS control mice in this study is in accordance with previous reports [18,47], although with slightly different order of magnitude.

In addition to that, a more precise analysis of behavioral impact of ERT would have been achieved if animals were tested both at the beginning and at the end of treatment and these values were compared. However, as behavioral outcome was not our main goal, animals were tested only once, at the end of the treatment. Therefore, this high variability which couldn’t be explained by GAG cortex levels, cathepsin D, heart function or antibody levels was found in the behavior of treated animals and needs to be further studied. Nonetheless, intermediary results from the three parameters analyzed for locomotion (crossings, distance and velocity) are in accordance with intermediary levels of heart function, cathepsin D, activated glia and reduction of TNF-α on cortex.

It is important to acknowledge the limitations of working with an animal model to directly transpose conclusions to human beings. The effects seen on open field tests can be altered by improved function of other organs and not exclusively CNS. Also, the enzyme delivery method applied in mice models is different from what is done in patients. The use a bolus injection instead of a long infusion could result in a different biodistribution and better perfusion to the CNS.

In conclusion, our results suggest that, even if started late, ERT is effective in reducing urinary GAG and storage in visceral organs. Although heart GAG levels were also normalized and valve thickness was greatly reduced, heart function was not completely restored and there was no effect in aortic wall width. However, since cardio-respiratory complications are the main cause of death in Hurler patients, the improvements seen in this study could have important repercussions on patients’ quality of life. A further discussion on the ethical implications of ERT in severely mentally impaired patients, though, must be conducted but is out of the scope of the present study The introduction of ERT, even later in life, can have beneficial effects on many aspects of the disease and should be considered whenever possible.

## Supporting Information

S1 Fig Experimental design. Control groups: 8 months normal (Idua^+/+^ or Idua^+/-^, n = 8) and MPS I (n = 11) mice. Treated group: MPS I mice treated from 6 to 8 months of age (ERT 6–8mo, n = 10) with 1.2mg laronidase/kg every 2 weeks.
(TIF)

S2 Fig Antibody formation. Serum IgG Anti-laronidase antibodies of 8-month old normal (n = 4), MPS I (n = 3) and 6 to 8 months laronidase treated MPS I (ERT 6–8mo, n = 10), 2 weeks after last injection. Each symbol represents one animal. Normal and MPS I serum were diluted 1:50 and ERT, 1:250. *P<0.001, ANOVA and Tukey *post hoc*.
(TIF)

S1 Table Comparison of neonatal, adult and late ERT outcomes. MPS I mice were treated with 1.2mg/kg of laronidase intravenously every two weeks for the indicated periods.
(DOCX)
