# Supplementary figures and images for: Effects of Enzyme Replacement Therapy Started Late in a Murine Model of Mucopolysaccharidosis Type I
Source: PLoS One. 2015 Feb 3;10(2):e0117271. doi: 10.1371/journal.pone.0117271 (PMC4315431; doi:10.1371/journal.pone.0117271)

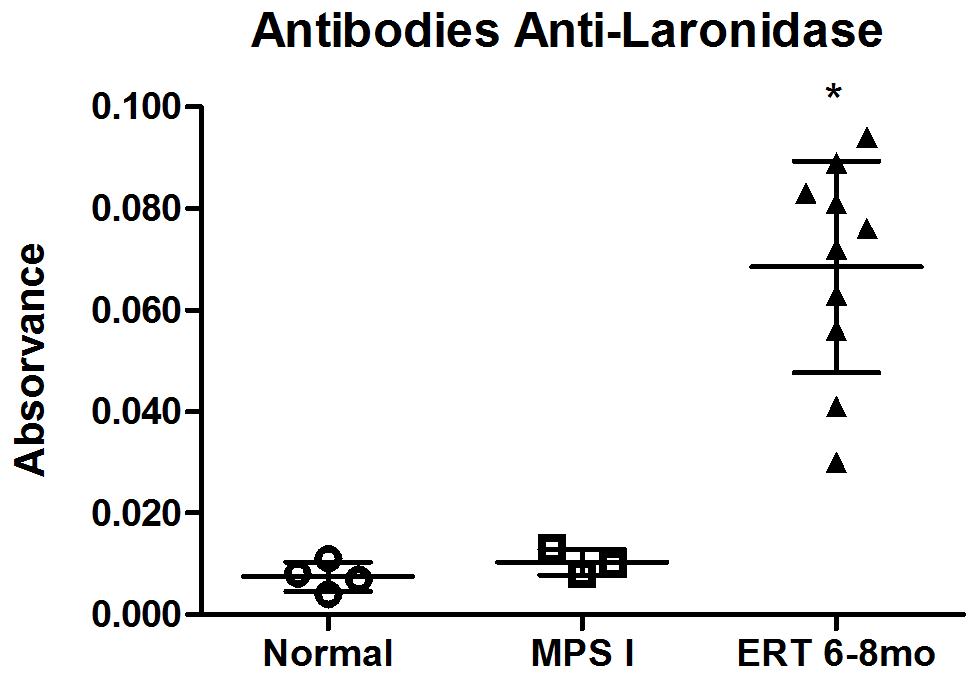

Supplement: S2 Fig — Serum IgG Anti-laronidase antibodies of 8-month old normal (n = 4), MPS I (n = 3) and 6 to 8 months laronidase treated MPS I (ERT 6–8mo, n = 10), 2 weeks after last injection. Each symbol represents one animal. Normal and MPS I serum were diluted 1:50 and ERT, 1:250. *P<0.001, ANOVA and Tukey post hoc. (TIF) [file pone.0117271.s002.tif]
