# Supplementary material for: Effects of Enzyme Replacement Therapy Started Late in a Murine Model of Mucopolysaccharidosis Type I
Source: PLoS One. 2015 Feb 3;10(2):e0117271. doi: 10.1371/journal.pone.0117271 (PMC4315431; doi:10.1371/journal.pone.0117271)
Supplement: S1 Table — MPS I mice were treated with 1.2mg/kg of laronidase intravenously every two weeks for the indicated periods. (DOCX) [file pone.0117271.s003.docx]

| **Symptons reversal after Treatment** | | | | |
| --- | --- | --- | --- | --- |
| **Group** | | **Neo ERT** [1] | **Adult ERT** [1] | **Late ERT** |
| **Length of ERT treatment** | | 0 to 6 months | 2 to 6 months | 6 to 8 months |
| GAGs levels | Urine | Yes | Yes | Yes |
|  | Liver | Yes | Yes | Yes |
|  | Kidney | Yes | Yes | Yes |
|  | Lung | Yes | Yes | Yes |
|  | Heart | Yes | Yes | Yes |
|  | Cerebral cortex | Yes | Yes | No |
| Heart function | LV Shortening Fraction | Yes | Yes | Yes |
|  | LV Ejection Fraction | Yes | Yes | Partly |
|  | AT/ET Ratio - pulmonary valve | Yes | Yes | Partly |
|  | Heart valves thickness | Yes | Partly | Partly |
|  | Aortic wall thicness | Yes | Partly | Partly |
| Open Field | Crossings | Yes | Yes | Partly |
|  | Rearings | Yes | Yes | No |
| Inflammatory markers in cerebral cortex | Cathepsin D activiy | Yes | Yes | Partly |
|  | GPAF staining | Yes | Yes | Partly |
|  | MIP 1-alpha expression | - | - | No |
|  | TNA alpha expression | - | - | Yes |

Reference:

1. Baldo G, Mayer FQ, Martinelli BZ, de Carvalho TG, Meyer FS, et al. (2013) Enzyme replacement therapy started at birth improves outcome in difficult-to-treat organs in mucopolysaccharidosis I mice. Mol Genet Metab 109: 33–40. doi:10.1016/j.ymgme.2013.03.005.
